# Publisher Correction: Nonlinear mechanics of human mitotic chromosomes

**DOI:** 10.1038/s41586-022-05051-y

**Published:** 2022-07-07

**Authors:** Anna E. C. Meijering, Kata Sarlós, Christian F. Nielsen, Hannes Witt, Janni Harju, Emma Kerklingh, Guus H. Haasnoot, Anna H. Bizard, Iddo Heller, Chase P. Broedersz, Ying Liu, Erwin J. G. Peterman, Ian D. Hickson, Gijs J. L. Wuite

**Affiliations:** 1 Department of Physics and Astronomy and LaserLaB Amsterdam, Vrije Universiteit Amsterdam, Amsterdam, The Netherlands; 2 Center for Chromosome Stability and Center for Healthy Aging, Department of Cellular and Molecular Medicine, University of Copenhagen, Copenhagen, Denmark

**Keywords:** Biological fluorescence, Supramolecular assembly, Thermodynamics, Single-molecule biophysics

Correction to: *Nature* 10.1038/s41586-022-04666-5 Published online 4 May 2022

In the version of this article initially published, Extended Data Fig. 5 was a duplicate of Extended Data Fig. 6. The correct image is now in place in the HTML and PDF versions of the article.

