# Supplementary material for: Nonlinear mechanics of human mitotic chromosomes
Source: Nature. 2022 May 4;605(7910):545–50. doi: 10.1038/s41586-022-04666-5 (PMC9117150; doi:10.1038/s41586-022-04666-5)
Supplement: Supplementary file 1 — This file contains Supplementary Notes 1 and 2, and Supplementary Figure 1. The Supplementary Notes contain a detailed description of the HWLC model and a discussion of selection bias on condensed chromosomes. Supplementary Figure 1 contains raw images of gels. [file 41586_2022_4666_MOESM1_ESM.pdf]

nature portfolio

https://doi.org/10.1038/s41586-022-04666-5

Supplementary information

Nonlinear mechanics of human mitotic chromosomes

In the format provided by the authors and unedited

Supplementary information for the article

"Nonlinear mechanics of human mitotic chromosomes"

Anna E.C. Meijering[1*], Kata Sarlós[2*], Christian F. Nielsen[2*], Hannes Witt[1], Janni Harju[1], Emma Kerklingh[1], Guus H. Haasnoot[1], Anna H. Bizard[2], Iddo Heller[1], Chase P. Broedersz[1,#], Ying Liu[2], Erwin J.G. Peterman[1**], Ian D. Hickson[2**,#], Gijs J.L. Wuite[1**,#]

*,** Shared authors
# Corresponding authors

[1]Department of Physics and Astronomy and LaserLaB Amsterdam, Vrije Universiteit Amsterdam, 1081 HV Amsterdam, the Netherlands
[2]Center for Chromosome Stability and Center for Healthy Aging, Department of Cellular and Molecular Medicine, University of Copenhagen, Blegdamsvej 3B, 2200 Copenhagen N, Denmark

SUPPLEMENTARY NOTES

Supplementary Note 1: Hierarchical Worm-Like Chain (HWLC) model

1. Introduction

To provide insight into the nonlinear mechanical response of a chromosome, we here develop and analyze a class of Hierarchical Worm-Like Chain (HWLC) models describing a heterogeneous serial assembly of nonlinear mechanical elements. Each of these assembly elements is characterized by its linear stiffness and critical force; the characteristic force threshold at which the element stiffens dramatically. To capture the heterogeneous nature of the chromosome, we assumed that the characteristics of the nonlinear elements in the assembly are sampled from given distributions. We propose that broad distributions of the mechanical characteristics of these elements define structurally heterogeneous models, which describe the integrated mechanical properties of complex assemblies, such as chromosomes.

Our aim was to predict the nonlinear mechanical response of a heterogeneous assembly, and to investigate if this expected response can quantitatively reproduce the anomalous stiffening behavior of the chromosomes we observed in our tweezer experiments.

2. Model for flexible WLC elements

Consider N elements extending in series, with component i characterised by its critical force $f_{c,i}$ and linear response stiffness $k_{0,i}$. These elements can correspond to segments of the chromosome with different elastic properties, to cross-linking structural components within a gel-like network experiencing shear, or to collective mechanical modes on different length-scales of a hierarchical assembly.

We assume that the critical forces are independently and identically distributed according to some probability density function $P(f_c)$. In general, $f_{c,i}$ and $k_{0,i}$ may not be independent, and they may follow some constitutive law $k_0(f_c) = \int P\,(k_0 \mid f_c) k_0 \mathrm{d}k_0$; the expected linear spring coefficient is a function of the element's critical force f_c. Here, we describe each element as a flexible worm-like chain with its contour length l_i larger than its persistence length $l_{p,i}$. We will discuss how our model generalizes to other element types in Section 3.

When a force F is exerted on the assembly, each flexible WLC element's extension x_i satisfies $F = g(x_i, f_{c,i}, k_{0,i})$, where[26]

$$g(x_i, f_{c,i}, k_{0,i}) = f_{c,i}\left(\frac{x_i}{l_i} - \frac{1}{4} + \frac{1}{4(1-x_i/l_i)^2}\right) \qquad \text{(S1)}$$

with $l_i = \frac{3}{2}\frac{f_{c,i}}{k_{0,i}}$. Note that in this case, since $k_0 \propto l^{-1} f_c$, we have that $k_0(f_c) \propto f_c \int P\,(l|f_c) l^{-1} \mathrm{d}l$, so that the constitutive law is set by possible correlations between the component length l_i and persistence length $l_{p,i} = k_B T f_{c,i}^{-1}$, with Boltzmann's constant k_B and temperature T.

The force response of the assembly is found by considering its total extension, $X = \sum_{i=1}^{N} x_i$. Thus, we have

$$F' := \frac{\mathrm{d}F}{\mathrm{d}X} = \left(\frac{\mathrm{d}X}{\mathrm{d}F}\right)^{-1} = \left(\sum_{i=1}^{N} \frac{1}{\mathrm{d}g(x_i, f_{c,i}, k_{0,i})/\mathrm{d}x_i}\right)^{-1}. \qquad \text{(S2)}$$

The dominant contributions to the total response $F'(F)$ will hence be given by the softest elements, with the smallest individual responses $\frac{\mathrm{d}g(x_i, f_{c,i}, k_{0,i})}{\mathrm{d}x_i}$. Geometrically, this implies that the total response curve of the assembly roughly follows the behaviour of the lowest individual response curve. This idea is illustrated by the total response of an assembly with three components, as shown in Extended Data Fig. 7a. The step-like features in the overall response curve are a result of the small number of elements in this example, and these features are expected to become less apparent as the number of elements increases.

For a general assembly, we distinguish two cases:

1. The individual response curves of the elements can cross. Consequently, the dominant element in the overall response of the assembly varies as a function of the force (Extended Data Fig. 7a). This corresponds to a constitutive law $k_0(f_c) \propto f_c^{\alpha}$, with $\alpha < 3/2$.
2. The individual response curves of the elements do not cross, implying that the element with minimum k_0 always dominates the stiffening response of the whole assembly (Extended Data Fig. 7b). This corresponds to a constitutive law $k_0(f_c) \propto f_c^{\alpha}$, with $\alpha > 3/2$.

In case 1, we can estimate the average total response of the chain by considering contributions from only those elements that are still in the linear response regime: $F'(F)^{-1} \approx \int_F^{\infty} \frac{NP(f_c=f)}{k_0(f)} \mathrm{d}f$. Note, this integral scales with $F^{-\gamma}$ if the integrand scales with $f^{-\gamma-1}$. Importantly, we see that our model predicts that the assembly stiffens according to a power-law relationship $F'(F) \propto F^{\gamma}$, if we assume a constitutive law $k_0(f) \propto f^{\alpha}$, together with a critical force distribution $P(f_c) \propto f_c^{-\beta}$, such that $\gamma = \alpha + \beta - 1$. We verified this scaling prediction by performing simulations of such a heterogenous assembly (Extended Data Fig. 7c)). In fact, by suitable choices of the parameters that specify the constitutive relation and the critical force distribution, we may construct an assembly in line with the experimentally measured chromosome response: $F'(F) \propto F^{\gamma}$, with $\gamma \approx 0.82$ (Fig. 3 d).

In case 2, individual response curves of the different components lie parallel to one another or asymptotically approach the same limit, and we therefore cannot neglect contributions to the total response from elements that have already started stiffening, as in case 1. For case 2, the expected total response can hence be approximated as $F'(F)^{-1} \approx \int_F^{\infty} \frac{NP(f_c=f)}{k_0(f)} \mathrm{d}f + \int_0^F \frac{NP(f_c=f)}{dg(x,f,k_0(f))/dx} \mathrm{d}f$. In general, no exact scaling behavior for the total stiffening response is expected, since the largest contributions come from the second term, and $\mathrm{d}g\,(x, f, k_0(f))/\mathrm{d}x$ scales differently at different f.

However, certain long-tailed distributions of $f_{c,i}$ can give stiffening responses which resemble scaling behavior. An example of this is the exponential distribution (Extended Data Fig. 7d).

3. **Extension to semi-flexible WLCs and other chain models**

Next, we demonstrate that the model described above can also be generalized to assemblies of other kinds of components, such as semi-flexible WLCs. This presupposes that each component's individual response follows $F = \tilde{g}(x_i, f_{c,i}, k_{0,i})$ for some $\tilde{g} \neq g$. As an example, for semi-flexible WLCs with $l_{p,i} > l_i$, we have[27]

$$\tilde{g}(x_i, f_{c,i}, k_{0,i}) = \frac{9 f_{c,i}}{\pi^2}\left(\frac{1}{(1-\epsilon_i)^2} - \frac{\epsilon_i}{3} - 1\right), \qquad \text{(S3)}$$

where $\epsilon_i = x_i \frac{\pi^2}{15} \frac{k_{0,i}}{f_{c,i}}$. In this limit, the constitutive law is given by $k_0(f_c) \propto f_c^2$.

The two categories of models described in Section 2 are further generalized by comparing the exponent of the constitutive law to the large-force stiffening behavior of each element. Specifically, if the elements follow a constitutive law $k_0(f_c) \propto f_c^{\alpha}$ and $\lim_{F \gg f_c} F'(F) \propto F^{\delta}$, the individual response curves of the elements cross when $\alpha < \delta$. This implies, for example, that all models consisting of semi-flexible chains, with $2 = \alpha > \delta = 3/2$, fall under category 2; the individual response curves never cross. As for flexible WLCs with $\alpha > 3/2$, serial assemblies of semi-flexible WLCs can exhibit stiffening behavior that resembles scaling, given that the critical forces are broadly distributed. As in the flexible case, an exponential distribution of critical forces can result in such behavior (Extended Data Fig. 7d).

4. **Choice of parameters for Figure 3**

To define a HWLC model, distributions for f_c, k_0, as well as the total number of elements, N, must be chosen. In general, many such choices can lead to similar average stiffening behaviour. Nevertheless, we wished to test whether reasonable parameter choices would allow us to reproduce the stiffening behaviour observed for chromosomes. For simplicity, we chose to fix N and L, and to assume that all elements were flexible WLCs with the same length, leading to the constitutive law $k_0 \propto f_c$. Despite these restrictions, a range of parameters was found to roughly fit the data for both the power-law and exponential distribution for f_c. To illustrate, a larger value of L, moving the stiffening curves down, as $K_0 \propto L^{-1}$ (Extended Data Fig. 3b/e) could be compensated for by increasing N (Extended Data Fig. 3a/d) and to some extent by varying the cut-offs for the distribution of f_c (Extended Data Fig. 3cf), data for upper cut-off not shown).

The lower cut-off for each distribution was set at the experimentally observed F_c, after which N and L were set to roughly match the variance and mean of the stiffening response. A value of $N \geq 10$ was chosen to ensure that most individual response curves did not have step-like features. The upper cut-off for the f_c distribution, as well f_c^* for the exponential distribution, were used to ensure that the anomalous stiffening behaviour persisted through the experimentally observed force range. We found that reasonable values of N and L could be used to fit the average stiffening curve using either distribution, while the variance was better described by the power-law distribution (Fig. 3de). Indeed, for the exponential distribution with $N \geq 10$, the variance at low forces remained lower than observed in experiment, and for both the exponential and the power-law distribution, the variance could not be matched in the high force regime by varying N (Extended Data Fig. 3ad). This suggests that variation in the assembly length L and/or the element length l could play a role in explaining the variance in the stiffening behaviour of chromosomes.

Supplementary Note 2: Selection bias on condensed chromosomes

The force-extension curves of the TOP2A-depleted chromosomes are qualitatively very similar to those for control chromosomes. We only observed a small change of the stiffening length from $2.8 \pm$

$0.3\ \mu m$ to $3.0 \pm 0.1\ \mu m$ (p=0.03) for the majority of the chromosomes (Fig. 4a and Extended Data Fig. 5a). However, an additional population of much longer chromosomes (with a stiffening length larger than 5 μm) was observed, as expected based on the observation of the hypo-condensed morphology in chromosome spreads (Extended Data Fig. 4d). In the tweezers, these longer chromosomes occurred less frequently than in the chromosome spread, which might be explained by a selection bias for the more contrast-rich, shorter chromosomes. Nevertheless, we observed that TOP2A-depleted chromosomes are slightly softer: their compliance (inverse stiffness) at 200 pN is significantly higher $3.5 \pm 0.4\ \mathrm{nm/pN}$ (TOP2A-depleted) versus $2.3 \pm 0.2\ \mathrm{nm/pN}$ (control), p=0.013 (Extended Data Fig. 5b). Moreover, the strain-stiffening power-law exponent is lower for TOP2A-depleted chromosomes (0.79 ± 0.04 (TOP2A-depleted) versus 0.98 ± 0.07 (control), p=0.04) (Extended Data Fig. 5c).

SUPPLEMENTARY FIGURES

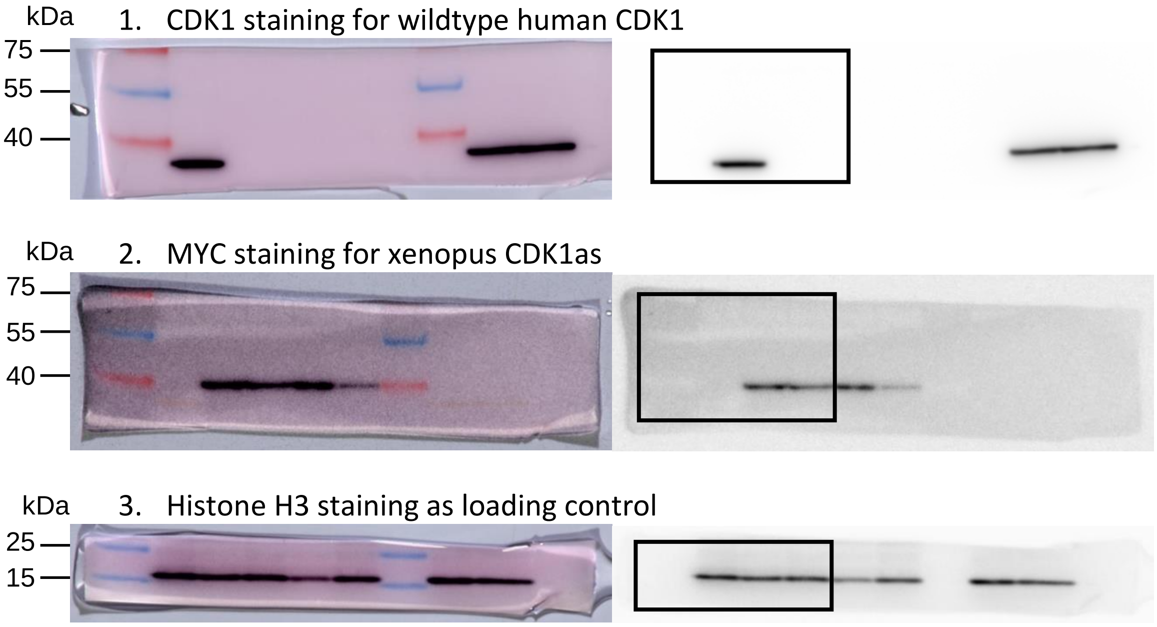

Supplementary Figure 1: Raw immunoblot images corresponding to Extended data Fig. 5A. The human CDK1 membrane (1.) was stripped and reblotted with anti-MYC antibody (2.). In the left images of 1. and 2. the size marker bands are, from the top, 70kDa (red), 55kDa (blue) and 40kDa (red). In the left images of 3., the size marker bands are, from the top, 25kDa and 15kDa (both blue). For Extended data Fig. 5A, the right-side images were cropped to contain the first four lanes (including marker) from the left, marked by black boxes.

SUPPLEMENTARY VIDEOS

Supplementary Video 1: Representative video from bright-field images of chromosome attachment to two microspheres using flow stretching.
Supplementary Video 2: Representative video from bright-field images of stretching a U2OS chromosome.
Supplementary Video 3: Representative video from bright-field images of three consecutive extension and retraction cycles of a U2OS chromosome.
Supplementary Video 4: Representative video from bright-field images of consecutive extension and retraction cycles of a HCT116 control chromosome. The first two extensions are at 80 mM KCl, after which the KCl concentration is alternated between 480 mM and 80 mM.
