# Supplementary material for: Nonlinear mechanics of human mitotic chromosomes
Source: Nature. 2022 May 4;605(7910):545–50. doi: 10.1038/s41586-022-04666-5 (PMC9117150; doi:10.1038/s41586-022-04666-5)
Supplement: Supplementary file 2 — Reporting Summary [file 41586_2022_4666_MOESM2_ESM.pdf]

nature portfolio

Corresponding author(s): Prof. Gijs Wuite
Prof. Ian Hickson

Last updated by author(s): Feb 4, 2022

Reporting Summary

Nature Portfolio wishes to improve the reproducibility of the work that we publish. This form provides structure for consistency and transparency in reporting. For further information on Nature Portfolio policies, see our Editorial Policies and the Editorial Policy Checklist.

Statistics

For all statistical analyses, confirm that the following items are present in the figure legend, table legend, main text, or Methods section.

n/a	Confirmed	
☐	☒	The exact sample size (*n*) for each experimental group/condition, given as a discrete number and unit of measurement
☐	☒	A statement on whether measurements were taken from distinct samples or whether the same sample was measured repeatedly
☐	☒	The statistical test(s) used AND whether they are one- or two-sided<br>*Only common tests should be described solely by name; describe more complex techniques in the Methods section.*
☒	☐	A description of all covariates tested
☒	☐	A description of any assumptions or corrections, such as tests of normality and adjustment for multiple comparisons
☐	☒	A full description of the statistical parameters including central tendency (e.g. means) or other basic estimates (e.g. regression coefficient) AND variation (e.g. standard deviation) or associated estimates of uncertainty (e.g. confidence intervals)
☐	☒	For null hypothesis testing, the test statistic (e.g. *F*, *t*, *r*) with confidence intervals, effect sizes, degrees of freedom and *P* value noted<br>*Give P values as exact values whenever suitable.*
☒	☐	For Bayesian analysis, information on the choice of priors and Markov chain Monte Carlo settings
☒	☐	For hierarchical and complex designs, identification of the appropriate level for tests and full reporting of outcomes
☒	☐	Estimates of effect sizes (e.g. Cohen's *d*, Pearson's *r*), indicating how they were calculated

Our web collection on statistics for biologists contains articles on many of the points above.

Software and code

Policy information about availability of computer code

Data collection	Custom made LabVIEW (2011 SP1) software was used to obtain optical tweezers data
Data analysis	Custom made MATLAB (2020a) scripts were used to analyse the data, simulations of HWLC were preformed using custom written Julia (1.6) scripts

For manuscripts utilizing custom algorithms or software that are central to the research but not yet described in published literature, software must be made available to editors and reviewers. We strongly encourage code deposition in a community repository (e.g. GitHub). See the Nature Portfolio guidelines for submitting code & software for further information.

Data

Policy information about availability of data

All manuscripts must include a data availability statement. This statement should provide the following information, where applicable:

- Accession codes, unique identifiers, or web links for publicly available datasets
- A description of any restrictions on data availability
- For clinical datasets or third party data, please ensure that the statement adheres to our policy

The data supporting the findings in this study are openly available from the DataverseNL repository at https://doi.org/10.34894/XFZZPJ.

Field-specific reporting

Please select the one below that is the best fit for your research. If you are not sure, read the appropriate sections before making your selection.

☒ Life sciences ☐ Behavioural & social sciences ☐ Ecological, evolutionary & environmental sciences

For a reference copy of the document with all sections, see nature.com/documents/nr-reporting-summary-flat.pdf

Life sciences study design

All studies must disclose on these points even when the disclosure is negative.

Sample size	No sample size calculations were performed. The sample sizes are indicated in the method section/figure captions and follow typical values used in similar micromechanical studies.
Data exclusions	Data was excluded if chromosomes detached during the experiment or if other experimental errors occurred such as air bubbles moving inside the flow chamber or experimental drift due to drying of water on the objective.
Replication	The experiments were performed spread over the course of more than one year, measured on more than 5 different biologically independent samples and performed by different experimenters showing consistent results. All replications were successful except for the exclusion criteria detailed above.
Randomization	This does not apply in our case since the experimental protocol inherently guarantees random selection of chromosomes.
Blinding	No blinding was applied during data collection or analysis. However, standardized procedures for data collection and analysis were used to prevent bias.

Reporting for specific materials, systems and methods

We require information from authors about some types of materials, experimental systems and methods used in many studies. Here, indicate whether each material, system or method listed is relevant to your study. If you are not sure if a list item applies to your research, read the appropriate section before selecting a response.

Materials & experimental systems

n/a	Involved in the study	
☐	☒	Antibodies
☐	☒	Eukaryotic cell lines
☒	☐	Palaeontology and archaeology
☒	☐	Animals and other organisms
☒	☐	Human research participants
☒	☐	Clinical data
☒	☐	Dual use research of concern

Methods

n/a	Involved in the study	
☒	☐	ChIP-seq
☒	☐	Flow cytometry
☒	☐	MRI-based neuroimaging

Antibodies

Antibodies used	Primary antibodies: Anti-NCAPH (1:100, HPA002647, Sigma Aldrich), CREST anti-sera (1:200 HCT-0100, Immuno-vision), anti-TRF2 (1:100, sc-9143, Santa Cruz), anti-H3S10 (1:400, 06-570, Sigma-Aldrich) and anti-H3-Alexa Fluor 647 (1:200, 15930862, ThermoFisher), anti-CDK1 (1:1000, ab133327, Abcam), anti-Myc (1:1000, sc-40, Santa Cruz) anti-histone H3.3 (1:5000, ab176840, Abcam).<br>Secondary antibodies: anti-rabbit IgG-Alexa Fluor 647 (1:200, A-21244, ThermoFisher), anti-rabbit IgG-Alexa Fluor 568 (1:200, A-11011, ThermoFisher) and anti-human IgG-Alexa Fluor 488 (1:200, A-11013, ThermoFisher), anti-mouse IgG peroxidase conjugate (1:10000, A4416, Sigma-Aldrich) and anti-rabbit IgG peroxidase conjugate (1:10000, A6154, Sigma-Aldrich).
Validation	HPA002647 (NCAPH) was validated by IHC and WB, and was used in the Human Protein Atlas and in Zhan et al. 2018 Oncol Rep and Scott et al. 2017 Mol Syst Biol. HCT-0100 antisera (CREST) was validated by ELISA and used in Nielsen et al. 2020 PNAS. sc-9143 (TRF2) was used in Liu at al. 2017 Cell and 26 other publications, but validation data is not available from the company. 06-570 (H3S10) was validated by ICC, WB and IP and used in Otsuki and Brand 2018 Science and more than 100 other publications. 15930862 (H3-AF 647) was validated by ICC/IF. ab133327 (CDK1) was validated by WB, IP and IHC-P, and used in Valeri-Alberni et al. 2021 Cell Rep and 43 other publications. Sc-40 (MYC) was validated by WB, IHC and IF, and published in Arany et al. 1993 Viral Immunol and Seo et al. 2021 Nat Comm. ab176840 (H3.3) was validated by ChIP, WB, IF and IHC and used in Udugama et al. 2021 Nat Comm and 16 other publications.<br>For details see manufacturer websites.

Eukaryotic cell lines

Policy information about cell lines

Cell line source(s)	The parental U2OS cells were obtained from and authenticated by ATCC (USA). They were modified into U2OS BirA-TRF1 cells and authenticated like described in Garcia-Exposito et al. 2016, Cell reports. The parental HCT116 CMV-TIR1 cells [Natsume et al. 2016, Mol Cell] to the HCT116 TOP2A-mAID cells and obtained from and authenticated by RIKEN, BRC Cell bank in Tsukuba, Japan. The HCT116 CMV-TIR1 cells were modified into HCT116 TOP2A-mAID and HCT116 TOP2A-mAID H2B-EGFP cells and authenticated by PCR, Immunoblotting and Sanger sequencing like described in Nielsen et al. 2020, PNAS. HCT116 TOP2A-mAID and HCT116 TOP2A-mAID H2B-EGFP cells were routinely grown with triple antibiotic selection (blastidicin, hygromycin, puromycin) to retain purity. HEK293T cells were obtained from and authenticated by ATCC (USA).
Authentication	The ATCC authenticated the wild type U2OS, HCT116 and HEK293T cell lines used in this study by STR profiling.
Mycoplasma contamination	All cell lines were tested negative for mycoplasma contamination
Commonly misidentified lines (See ICLAC register)	No commonly misidentified cell line was used in the study.
